# Mutant knock-in mice display enhanced susceptibility to pure prion protein fibrils

**DOI:** 10.1099/jgv.0.002219

**Published:** 2026-02-05

**Authors:** Daniel J. Walsh, Heidi Standke, Allison Kraus, Joel C. Watts, Surachai Supattapone

**Affiliations:** 1 Department of Biochemistry and Cell Biology, Geisel School of Medicine at Dartmouth, Hanover, New Hampshire 03755, USA; 2 Department of Pathology, Case Western Reserve University School of Medicine, Cleveland, OH, USA; 3 Tanz Centre for Research in Neurodegenerative Diseases and Department of Biochemistry, University of Toronto, Toronto, Ontario, Canada; 4 Department of Medicine, Geisel School of Medicine at Dartmouth, Hanover, New Hampshire 03755, USA

**Keywords:** fatal familial insomnia, fCJD, fibril, genetic, mutant, prion

## Abstract

Prion diseases manifest clinically in three different forms. Sporadic and infectious forms of prion disease are caused by the conversion of WT, cellular prion protein (PrP^C^) into its pathogenic conformer (PrP^Sc^). In contrast, genetic forms of prion diseases are caused by mutations in the PrP sequence that promote mutant PrP^Sc^ formation. When reconstituted with either polyanionic or lipid cofactors, purified PrP^C^ substrate can be converted *in vitro* into PrP^Sc^ products that display high levels of specific infectivity when inoculated in WT hosts. In contrast, various protein-only PrP^Sc^ molecules formed in the absence of cofactors display much lower levels of specific infectivity. Here, we report that protein-only PrP^Sc^ molecules with different sequences can induce the formation of proteinase K-resistant PrP^Sc^ molecules and spongiform degeneration in the brains of knock-in mice expressing PrP harbouring the pathogenic E200K mutation, but not in hosts expressing WT PrP. These results indicate that the E200K mutation enhances host susceptibility to various protein-only PrP^Sc^ fibrils, suggesting fundamental differences in the replication mechanisms of WT versus mutant prions.

## Introduction

Prion diseases are fatal neurodegenerative disorders caused by the misfolding of the host-encoded prion protein [cellular prion protein (PrP^C^)] into an aggregated conformer (PrP^Sc^) [1,2]. Human cases fall into three broad categories – genetic, sporadic and infectious – which differ in origin, epidemiology and clinical features [3]. Genetic forms of disease such as familial Creutzfeldt–Jakob disease (fCJD), Gerstmann–Sträussler–Scheinker and fatal familial insomnia (FFI) are caused by pathogenic PrP mutations such as E200K in fCJD and D178N in FFI [4,5]. These mutations drive the formation of mutant PrP^Sc^ molecules and subsequent neurodegeneration in human patients. In contrast, sporadic and infectious forms of prion disease, such as sporadic CJD, scrapie, bovine spongiform encephalopathy and chronic wasting disease are caused by the auto-catalytic conversion of WT PrP^C^ into PrP^Sc^ by an unknown mechanism.

Our lab and others have replicated various synthetic PrP^Sc^ molecules (operationally defined here as protease-resistant PrP conformers that self-propagate faithfully and efficiently) *in vitro* with markedly different levels of specific infectivity. Collectively, these studies have shown that non-proteinaceous cofactors are required to produce WT prions with high levels of specific infectivity [6,11]. Interestingly, in the absence of cofactor molecules, both WT and mutant PrP substrates can also form self-replicating amyloid fibrils with PIRIBS (parallel in-register intermolecular β-sheet) architecture, but these ‘protein-only’ fibrils display relatively low levels of specific infectivity in hosts expressing WT PrP^C^ molecules without amyloidogenic polymorphisms or mutations, such as I109, D178N or E200K [7,11,15]. This is true even for protein-only PrP^Sc^ molecules seeded with the same template as fully infectious cofactor PrP^Sc^ molecules [7].

Knock-in (ki) mice expressing bank vole (BV) PrP^C^ molecules with the I109 polymorphism (kiBVI) have recently been developed [16]. The I109 polymorphism promotes the spontaneous formation of PrP^Sc^ in transgenic mice overexpressing I109 BV PrP [17], but kiBVI mice do not develop spontaneous disease. In contrast, ki mice expressing I109 BV PrP containing D178N and E200K mutations (kiBVI^D178N^ and kiBVI^E200K^, respectively) spontaneously develop prion disease associated with spongiform degeneration [16]. However, spontaneously sick kiBVI^D178N^ and kiBVI^E200K^ mice do not appear to accumulate classical forms of proteinase K (PK)-resistant PrP^Sc^ in their brains [16]. Because prior studies suggest that different mechanisms may be responsible for the propagation of WT and mutant prions (i.e. with PrP sequences containing pathogenic mutations such as D178N or E200K or amyloidogenic polymorphisms such as I109) [18,20], we sought to determine whether any of these new ki mouse lines might be more susceptible to various protein-only PrP^Sc^ molecules than WT hosts.

## Methods

### *In vitro* propagation of protein recPrP^Sc^ samples

Cocktails for protein-only D177N and E199K mouse (Mo) PrP^Sc^ serial propagation reactions were prepared as previously described [18]. Unseeded reactions containing 6 µg ml^−1^ purified Mo recPrP 23–230 (either D177N or E199K) in conversion buffer (20 mM Tris, 135 mM NaCl, 5 mM EDTA pH 7.5, 0.15% (v/v) Triton X-100, pH 7.4) without cofactors in a total volume of 400 µl were shaken in 1.5 ml microfuge tubes and serially propagated at a 20% (v/v) seeding ratio. Shaking was performed as previously described [19] in a home-built machine with an 8 mm orbit shaking at ∼1,200 r.p.m. Each reaction was shaken for 72 h at a temperature of 37 °C before propagation to the next round.

Cocktails for protein-only I109 BV serial propagation reactions containing 6 µg ml^−1^ purified I109 BV recPrP 23–230 in conversion buffer without cofactors were prepared and initially seeded with protein-only I109 BV protein-only PrP^Sc^ generated by protein misfolding cyclic amplification (PMCA) as previously described [11] and similarly shaken and serially propagated at 1,200 r.p.m. in our custom-built shaker, as described above for mutant PrP samples.

### Rodent models

CD-1 mice were purchased from Charles River Laboratories (Worcester, MA). BVs were kindly provided by Umberto Agrimi (Rome, Italy). The ki mouse models kiBVI (expressing I109 BV PrP), kiBVI^D178N^ (expressing I109 D178N BV PrP) and kiBVI^E200K^ (expressing I109 E200k BV PrP) used in this study have been previously described [16].

### Inoculation, diagnosis and tissue harvest

Intracerebral inoculation and diagnosis of prion disease were performed as described [11,21] with the following modifications. *In vitro*-converted mutant PrP^Sc^ samples were dispersed by three 20 s sonication pulses in a cup horn sonicator (Qsonica Misonix, Newtown, CT) and subsequently diluted tenfold into PBS+1% BSA to be used as inoculum (i.e. final concentration of PrP^Sc^=0.6 µg ml^−1^). The inoculum volume used was 30 µl. Rodents were inoculated between 4 and 6 weeks of age or allowed to age without intervention until the onset of scrapie. At terminal disease stage, mice were euthanized and brains were recovered. Brains for biochemical analysis were immediately frozen at −70 °C, and brains for histopathology were fixed in PBS+4% formalin.

### Proteinase K digestion of PrP^Sc^

Brain homogenates (10% (w/v) in PBS) from experimental mouse brains were digested in a reaction containing 10 µg ml^−1^ PK, 1% (v/v) Triton X-100, 0.5% sodium deoxycholate at 37 °C with shaking at 1000 r.p.m. for 1 h. PrP^Sc^ samples produced *in vitro* were treated with 20 µg ml^−1^ PK at 37 °C for 30 min. All digestion reactions were quenched by addition of 4 mM PMSF.

### ReadyBlue-stained SDS-PAGE

Aliquots of recombinant protein-only PrP^Sc^ samples produced *in vitro* were treated with 20 µg ml^−1^ PK at 37 °C for 30 min. All digestion reactions were quenched by the addition of 4 mM PMSF. Samples were centrifuged at 18,000 ***g*** for 1 h at 4 °C. Supernatants were discarded, and pellets were resuspended in ~60 µl total volume Lauryl dodecyl sulfate sample buffer (Invitrogen, Carlsbad, CA). All samples were boiled for 7 min, and 25 µl of each sample was loaded into a pre-cast 10% NuPage Bis-Tris gel (Invitrogen) alongside a molecular weight marker lane containing SeeBlue Plus 2 prestained standard (Invitrogen) and run in MES SDS running buffer (Invitrogen) at 200V for 35 min. The gel was subsequently stained with ReadyBlue Protein Gel Stain (Sigma Aldrich, St. Louis, MO) and imaged with an Azure 600 imager (Dublin, CA).

### Western blotting

All protein samples were denatured by boiling at 95 °C in Laemmli SDS sample buffer (Bioland Scientific) for 15 min. Denatured protein samples were run on 12% SDS-PAGE gels and transferred to an Immobilon-P PVDF membrane (Millipore, Burlington, MA) using a semi-dry blotting apparatus (Bio-Rad, Hercules, CA). Western blots were probed using anti-PrP D18 primary antibody (epitope 132–156)and HRP-linked goat anti-human secondary antibody (Invitrogen).

### Histopathology

Neuropathology was performed as previously described [22]. Formalin-fixed brains were disinfected by immersion in 96% formic acid for 1 h. Tissue blocks and 4 µm thick microscopic sections stained with haematoxylin and eosin were prepared by the Dartmouth Hitchcock Research Pathology Service Core (Lebanon, NH), and vacuolation in various brain regions was scored on a 0–5 scale.

### MALDI-TOF mass spectrometry

To prepare mutant Mo PrP^Sc^ conformers for mass analysis, 5 ml of converted cocktail was first treated with PK, as described above, and then quenched with PMSF. N-Octyl-β-d-glucopyranoside (Anatrace, Maumee, OH) detergent was added to a final concentration of 1% (w/v), and samples were end-over-end rotated for 30 min at room temperature to allow for full solubilization. After 1 h of centrifugation at 18,000 ***g***, the supernatant was discarded, and pellets were resuspended in an equal volume of water. This centrifugation and water wash step was then repeated to remove any residual buffer components. After a third centrifugation, pellets were denatured by resuspending in 100 µl of 6 M guanidine hydrochloride and incubating for 3 h at 60 °C, shaking at 900 r.p.m. After centrifugation again at 18,000 ***g*** for 1 h, denatured protein was isolated from the supernatants by methanol/chloroform precipitation [23]. Protein pellets from the interphase were dried and then resuspended in 30 µl water.

MALDI-TOF mass spectrometry was performed as described previously [24]. Briefly, the protein sample was concentrated using an OMIX C4 chromatographic pipette tip (Varian Inc., Santa Clara, CA) and eluted with sinapinic acid matrix onto a Voyager 100-position MALDI sample plate (Applied Biosystems, Foster City, CA). Spectra were acquired using a Voyager-DE Pro Biospectrometry Workstation (Applied Biosystems) with a mass accuracy of ±10 Da.

### Electron microscopy

To prepare D177N recPrP^Sc^ for negative stain imaging, n-octyl-β-d-glucopyranoside was added to shaken cocktails at 1% (w/v) to solubilize unconverted protein, and solutions were centrifuged at 4,000 ***g*** to collect fibrillar material. Pellets were subjected to two additional centrifugation and wash steps with 1/3× PBS, 0.02% amphipol to remove detergent and concentrate the sample for application to electron microscopy grids.

Four hundred mesh lacey carbon grids (Ted Pella, Redding, CA) were glow discharged and placed on a 7 µl droplet of concentrated D178N PrP^Sc^ sample and incubated for 20 min in a humidified chamber. Grids were then blotted on filter paper, washed in Nano-W stain (Nanoprobes, Yaphank, NY), blotted again and then stained by placing on a droplet of Nano-W for 1 min. Grids were then blotted dry and imaged on an FEI (Field Electron and Ion Company) Tecnai TF20 (200 kV, FEG) with a 4k × 4k CMOS (Complementary Metal-Oxide-Semiconductor)-based Tietz TemCam-F416 camera.

## RESULTS

We first inoculated three different preparations of protein-only PrP^Sc^ molecules into WT hosts and kiBVI mice. I109 BV protein-only PrP^Sc^, D177N Mo protein-only PrP^Sc^ and E199K Mo protein-only PrP^Sc^ (note D177N and E199K in Mo PrP correspond to D178N and E200K BV and human PrP) were generated as previously described [11,18] and propagated by shaking in the absence of cofactor molecules [19]. CD-1 mice, WT BVs and kiBVI mice all remained healthy for >600 days following inoculation with any of the three protein-only PrP^Sc^ preparations (Table 1). Next, we inoculated the same three protein-only PrP^Sc^ preparations into kiBVI^D178N^ and kiBVI^E200K^ mice. As expected, uninoculated kiBVI^D178N^ and kiBVI^E200K^ control mice developed spontaneous disease (Table 2). Inoculation of various protein-only PrP^Sc^ molecules did not cause statistically significant changes in disease-free survival in either line of ki mice, with one exception: inoculation of D177N Mo protein-only PrP^Sc^ into kiBVI^E200K^ mice reduced disease-free survival from ~650 days to ~340 days (*P*-value=0.0003) (Table 2).

**Table 1.** Bioassay of protein-only PrP^Sc^ molecules in rodents expressing various WT PrP sequences

Host	Inoculum	n/n_0_	IP (days)^*^
CD-1 mice	Negative control	0/8	>700
	E199K Mo protein-only PrP^Sc^	0/4	>700
	D177N Mo protein-only PrP^Sc^	0/4	>700
M109 bank voles	Negative control	0/8	>700
	I109 BV protein-only PrP^Sc^	0/3	>700
	E199K Mo protein-only PrP^Sc^	0/4	>700
	D177N Mo protein-only PrP^Sc^	0/7	>700
kiBVI mice	Negative control	0/5	>650
	I109 BV protein-only PrP^Sc^	0/4	>650
	E199K Mo protein-only PrP^Sc^	0/7	>600
	D177N Mo protein-only PrP^Sc^	0/10	>600

*Mean incubation period (IP) of scrapie sick animals+standard error.

**Table 2.** Bioassay of protein-only PrP^Sc^ molecules in ki mice expressing mutant PrP sequences

Host	Inoculum	n/n_0_	IP (days)^*^
kiBVI^D178N^ mice	Negative control	7/7	518+21
	I109 BV protein-only PrP^Sc^	4/4	466+85
	E199K Mo protein-only PrP^Sc^	6/6	485+26
	D177N Mo protein-only PrP^Sc^	6/6	499+8
kiBVI^E200K^ mice	Negative control	8/8	646+70
	I109 BV protein-only PrP^Sc^	3/3	625+37
	E199K Mo protein-only PrP^Sc^	5/5	603+46
	D177N Mo protein-only PrP^Sc^	9/9	336+23†

*Mean incubation period (IP) of scrapie sick animals+standard error.

†*P*-value=0.0003 compared to negative control, determined by 1-tail, type 3 t-test.

**Fig. 1.**
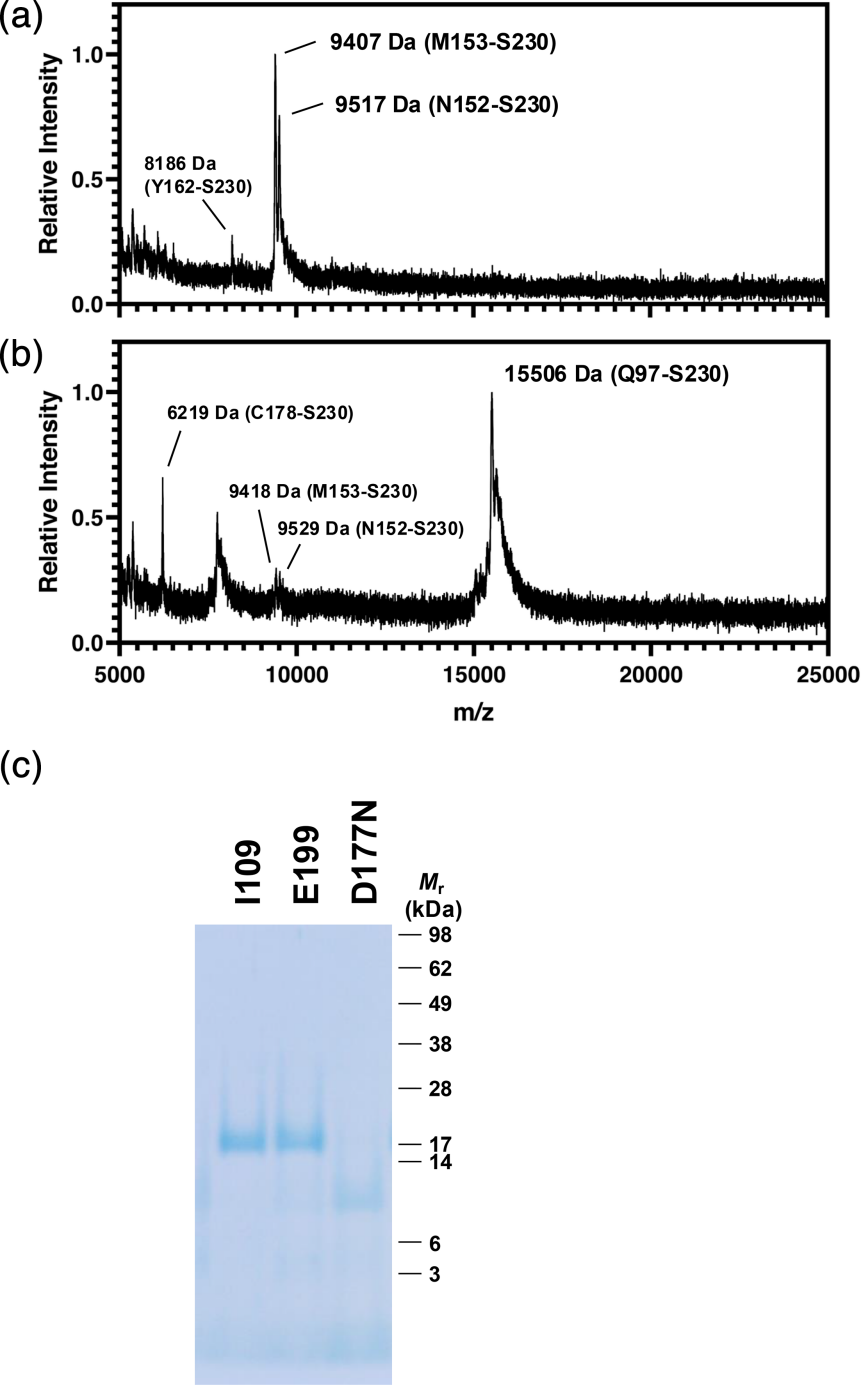
Mass analysis of protease-resistant domains in mutant PrP^Sc^ conformers. MALDI-TOF mass spectra of (**a**) D177N and (**b**) E199K Mo PrP^Sc^ conformers after digestion with proteinase K. Peaks unambiguously assigned are labelled with both the mass and the identity of the fragment. Unlabelled peaks either could not be confidently assigned or represent the z=2 charge state of the majority fragment. (**c**) ReadyBlue-stained SDS-PAGE of I109 BV, E199K Mo and D177N Mo protein-only PrP^Sc^ samples following limited proteolysis with proteinase K.

**Fig. 2.**
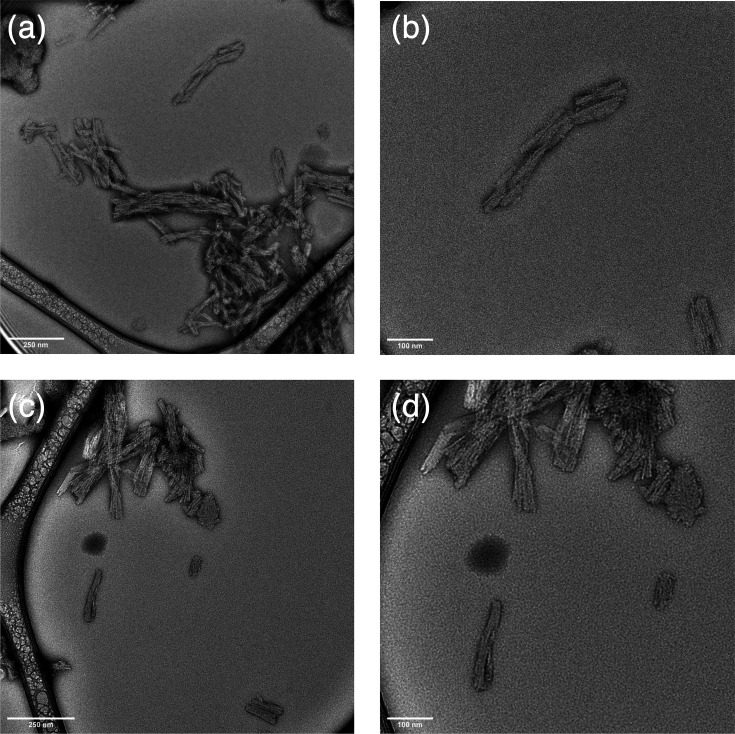
D177N recPrP^Sc^ assembles into fibrillar aggregates. Negative stain electron micrographs of shaken D177N recPrP^Sc^ fibrils at varying magnification. Scale bars are 250 nm (**a, c**) and 100 nm (**b, d**).

Interestingly, the PK-resistant core of D177N Mo protein-only PrP^Sc^ is much smaller than that of other cofactor and protein-only PrP^Sc^ conformers [7,11, 14, 15, 18, 19]. Using MALDI-TOF mass spectrometry, we determined that the PK-resistant core of D177N Mo protein-only PrP^Sc^ encompasses residues 152–230 (Fig. 1a); for comparison, the PK-resistant core of E199K Mo protein-only PrP^Sc^ is 97–230 (Fig. 1b). It should be noted that both E199K and D177N PrP^Sc^ molecules can generate both PK-resistant core sizes. In serial PMCA reactions, the relative amounts of the two sizes are similar [18], whereas in continuous shaking reactions, the relative distributions skew towards E199K PrP^Sc^ having predominantly the larger core and D177N PrP^Sc^ having predominantly the smaller core, but both sizes of PK-resistant bands remain visible for both conformers by SDS-PAGE (Fig. 1c) and Western blot [19]. Despite the relatively small size of its predominant protease-resistant core, D177N Mo protein-only PrP^Sc^ forms amyloid fibrils similar to a variety of other PrP^Sc^ conformers with larger protease-resistant cores [15,25,31], as determined by negative-stain electron microscopy (Fig. 2).

**Fig. 3.**
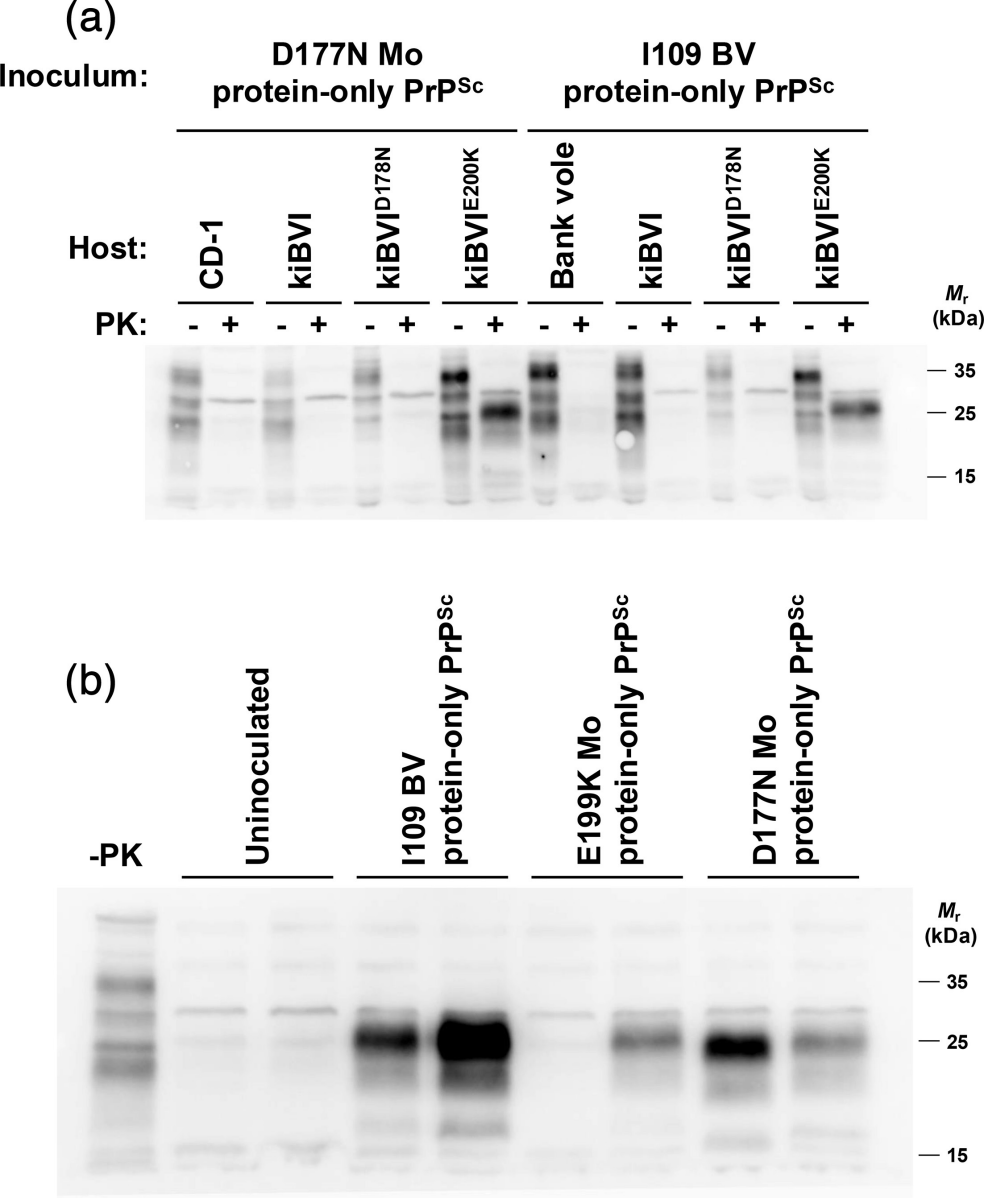
Western blots of proteinase K-resistant PrP^Sc^ molecules in the brains of experimental animals. (**a**) Brain homogenates prepared from various rodent hosts inoculated with either D177N Mo protein-only PrP^Sc^ or I109 BV protein-only PrP^Sc^, as indicated. PK=proteinase K digestion. (**b**) Brain homogenates prepared from kiBVI^E200K^ mice inoculated with various protein-only PrP^Sc^ preparations, as indicated. Samples taken from two independent animals are shown for each inoculum. -PK=sample not digested with proteinase K.

To test whether protein-only PrP^Sc^ inocula induced formation of new PrP^Sc^ molecules in the brains of host animals, we digested experimental brain homogenates with PK and visualized PK-resistant PrP^Sc^ molecules with Western blotting (Fig. 3). The results show that both D177N Mo protein-only PrP^Sc^ and I109 BV protein-only PrP^Sc^ induced formation of PK-resistant PrP^Sc^ in the brains of kiBVI^E200K^ mice, but not in WT mice or voles, kiBVI mice or kiBVI^D178N^ mice (Fig. 3a). PK-resistant PrP^Sc^ could also be detected in the brains of some but not all kiBVI^E200K^ mice inoculated with E199K Mo protein-only PrP^Sc^ (Fig. 3b). Taken together, these results suggest that kiBVI^E200K^ mice are a relatively good host for protein-only PrP^Sc^ inocula.

**Fig. 4.**
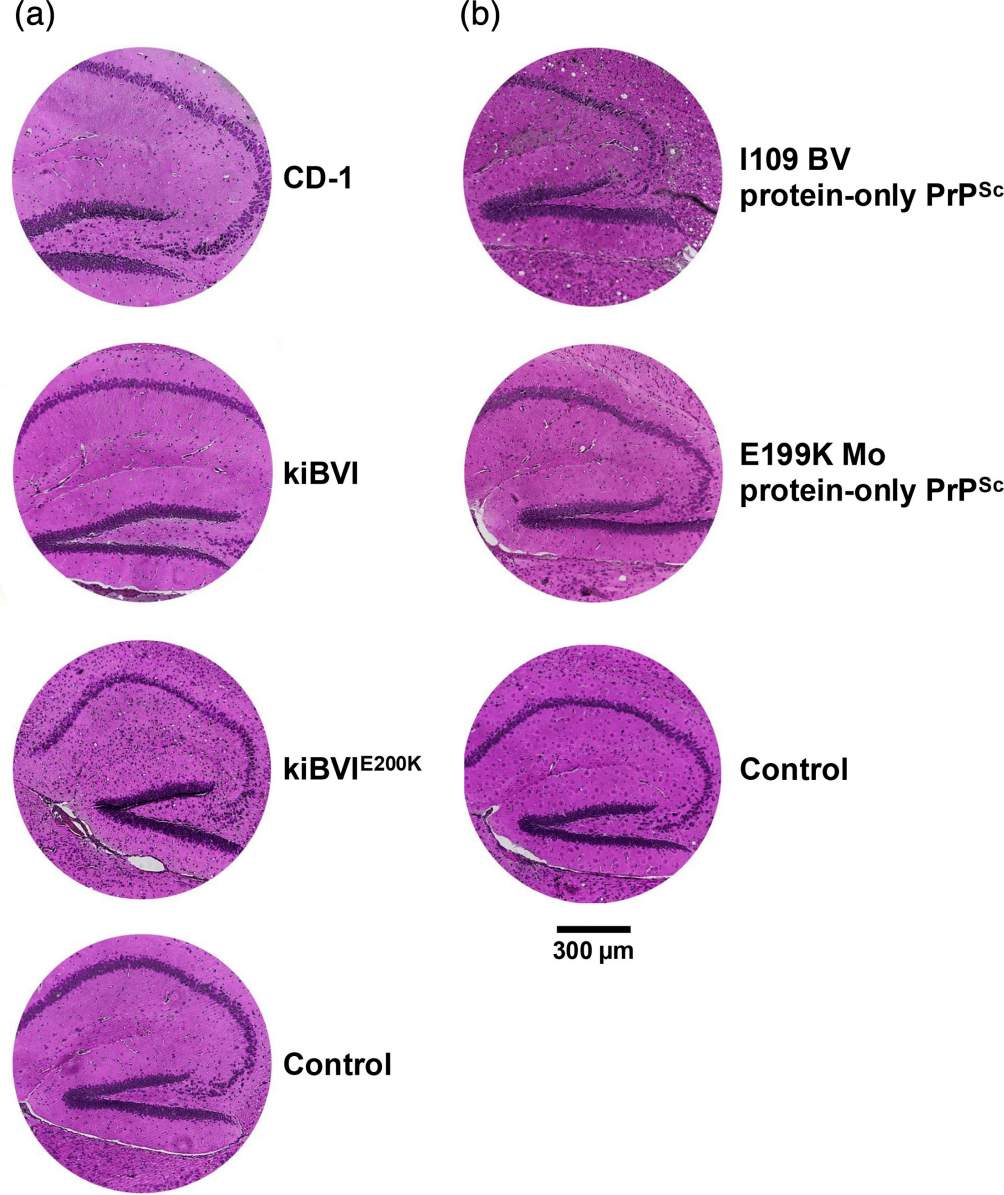
Neuropathology of rodents inoculated with protein-only PrP^Sc^ inocula. Representative microscopic images of the hippocampus in brain sections stained with haematoxylin and eosin (H and E) (**a**) Various rodents inoculated with D177N Mo protein-only PrP^Sc^, as indicated. Asymptomatic CD-1 mouse=8 months old, asymptomatic kiBVI mouse=20 months old, symptomatic kiBVI^E200K^ mouse=8 months old. Control=Uninoculated kiBVI^E200K^ mouse (14 months old, asymptomatic). (**b**) kiBVI^E200K^ mice inoculated with alternative protein-only PrP^Sc^ inocula, as indicated. Symptomatic mouse inoculated with I109 BV protein-only PrP^Sc^=23 months old, symptomatic mouse inoculated with E199K Mo protein-only PrP^Sc^=19 months old. Control=Uninoculated kiBVI^E200K^ mouse (23 months old, asymptomatic).

It is known that kiBVI^E200K^ mice spontaneously develop scrapie clinical signs with spongiform vacuolation at ~20 months of age [16,20]. To evaluate whether protein-only PrP^Sc^ inocula induce any additional neuropathological changes in kiBVI^E200K^ hosts, we assessed vacuolation specifically within the hippocampus, a brain region that displays only minimum levels of vacuolation in spontaneously sick kiBVI^E200K^ mice [16,20]. The results show that D177N protein-only PrP^Sc^ molecules induce vacuolation in the hippocampus of kiBVI^E200K^ mice, but not in CD-1 or kiBVI mice (Fig. 4a, compare the third row to the top two rows). Inoculation of I109 BV protein-only PrP^Sc^ also induces vacuolation in the hippocampus of kiBVI^E200K^ mice (Fig. 4b, top row), but inoculation of E199K Mo protein-only PrP^Sc^ does not (Fig. 4b, second row). Overall, our results indicate that kiBVI^E200K^ mice are relatively good hosts for various protein-only PrP^Sc^ inocula, but some protein-only PrP^Sc^ molecules are more efficient than others at inducing the formation of mutant PrP^Sc^ molecules, vacuolation and clinical signs.

## DISCUSSION

In this manuscript, we report that kiBVI^E200K^ mice are far more susceptible to protein-only PrP^Sc^ molecules than either WT hosts or kiBVI mice. Previous *in vitro* studies showed that cofactor molecules facilitate the formation of WT PrP^Sc^ (even when seeded by mutant prions), whereas mutant PrP substrate molecules can spontaneously convert into PrP^Sc^ without cofactor molecules [18,20]. Together with the results of those studies, the *in vivo* data reported here suggest that WT and mutant PrP^Sc^ molecules replicate by distinct mechanisms.

Western blot data indicate that various protein-only PrP^Sc^ molecules can propagate in kiBVI^E200K^ mice, but not in kiBVI or kiBVI^D178N^ mice. Even though transgenic mice overexpressing I109 BV PrP develop spontaneous prion disease, our results indicate that addition of the pathogenic E200K mutation is important for susceptibility to protein-only PrP^Sc^ inocula. The apparent inability of kiBVI^D178N^ mice to replicate various protein-only PrP^Sc^ molecules, even D177N Mo protein-only PrP^Sc^, is most likely an artefact due to the lower PrP^C^ expression level in kiBVI^D178N^ mice (~60% lower) compared to kiBVI^E200K^ mice [16].

Our data indicate that kiBVI^E200K^ mice were most susceptible to D177N Mo protein-only PrP^Sc^ and least susceptible to E199K Mo protein-only PrP^Sc^. Notably, inoculation with D177N Mo protein-only PrP^Sc^ accelerates the onset of scrapie clinical signs, induces the formation of PK-resistant PrP^Sc^ and causes specific vacuolation in the hippocampus of kiBVI^E200K^ mice. In contrast, I109 BV protein-only PrP^Sc^ induces the formation of PK-resistant PrP^Sc^ and vacuolation but does not accelerate disease, and E200K Mo protein-only PrP^Sc^ induces the formation of PK-resistant PrP^Sc^ without causing either vacuolation or disease acceleration. The structural basis for the relative pathogenicity of various protein-only PrP^Sc^ molecules in kiBVI^E200K^ mice remains unknown, but our results show neither the I109 BV backbone nor the E200K mutation in PrP^Sc^ is necessary for efficient transmission. Surprisingly, the most pathogenic inoculum, D177N Mo protein-only PrP^Sc^, (1) has the lowest degree of homology to the PrP^C^ sequence expressed in kiBVI^E200K^ mice, and (2) has predominantly a much smaller PK-resistant, structured core compared to the other two inocula (in addition to a small amount of a larger PK-resistant core). Typically, prion transmissions are more efficient when there is (1) greater sequence similarity between the inoculated PrP^Sc^ and host PrP^C^ and (2) a larger PK-resistant core [32]. We infer that I109 BV protein-only PrP^Sc^ and E199K Mo protein-only PrP^Sc^ were not able to accelerate clinical disease because they were unable to induce the formation of host PrP^Sc^ as quickly as D177N Mo protein-only PrP^Sc^, relative to the regular lifespan of the host animals. Interestingly, FFI caused by the D178N mutation has much higher clinical penetrance than fCJD caused by the E200K mutation in human patients [33] despite D178N PrP^Sc^ having a smaller PK-resistant core than E200K PrP^Sc^ [34]. Future structural and functional studies of these three protein-only PrP^Sc^ conformers may help us better understand their differences in pathogenicity.

It remains unclear why mutant prions can be formed without cofactors. Structural analysis by X-ray crystallography [35], nuclear magnetic resonance [36] and molecular dynamics simulations [23,37, 38] has suggested that disease-associated mutations can disrupt salt bridge and hydrogen bonding networks present in the normal cellular conformation of the protein, PrP^C^, leading to a destabilization of the alpha helix-rich fold and increased propensity for misfolding. It is possible that this destabilization circumvents the role normally played by cofactor molecules during the formation of WT prions.

In summary, we report the first set of *in vivo* experimental results which support the hypothesis that hosts expressing mutant and polymorphic PrP^C^ molecules use a different mechanism than WT hosts to replicate prions.

## Abbreviations

BV: bank vole
fCJD: familial Creutzfeldt–Jakob disease
FFI: fatal familial insomnia
ki: knock-in
PK: proteinase K
PMCA: protein misfolding cyclic amplification
PrP^C^: cellular prion protein
PrP^Sc^: pathogenic conformation of the prion protein
